# Potential Biological Applications of Bio-Based Anacardic Acids and Their Derivatives

**DOI:** 10.3390/ijms16048569

**Published:** 2015-04-16

**Authors:** Fatma B. Hamad, Egid B. Mubofu

**Affiliations:** 1 Chemistry Department, Dar es Salaam University College of Education, P.O. Box 2329, Dar es Salaam, Tanzania; E-Mail: hamadfatma@yahoo.com; 2 Chemistry Department, University of Dar es Salaam, P.O. Box 35061, Dar es Salaam, Tanzania

**Keywords:** cashew nut shell liquid, anacardic acid, antibacterial, antitumor, antioxidant

## Abstract

Cashew nut shells (CNS), which are agro wastes from cashew nut processing factories, have proven to be among the most versatile bio-based renewable materials in the search for functional materials and chemicals from renewable resources. CNS are produced in the cashew nut processing process as waste, but they contain cashew nut shell liquid (CNSL) up to about 30–35 wt. % of the nut shell weight depending on the method of extraction. CNSL is a mixture of anacardic acid, cardanol, cardol, and methyl cardol, and the structures of these phenols offer opportunities for the development of diverse products. For anacardic acid, the combination of phenolic, carboxylic, and a 15-carbon alkyl side chain functional group makes it attractive in biological applications or as a synthon for the synthesis of a multitude of bioactive compounds. Anacardic acid, which is about 65% of a CNSL mixture, can be extracted from the agro waste. This shows that CNS waste can be used to extract useful chemicals and thus provide alternative green sources of chemicals, apart from relying only on the otherwise declining petroleum based sources. This paper reviews the potential of anacardic acids and their semi-synthetic derivatives for antibacterial, antitumor, and antioxidant activities. The review focuses on natural anacardic acids from CNS and other plants and their semi-synthetic derivatives as possible lead compounds in medicine. In addition, the use of anacardic acid as a starting material for the synthesis of various biologically active compounds and complexes is reported.

## 1. Introduction

**Figure 1:**
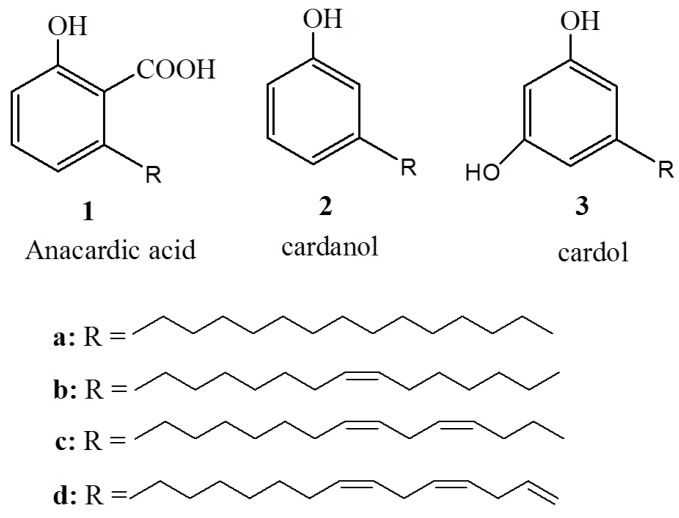
The major components of cashew nut shell liquid.

As part of cashew nut processing, the cashew nut shell (CNS) is released to the environment as an agricultural byproduct and waste. However, inside the soft honeycomb of the shell, there is a valuable greenish-yellow viscous liquid called cashew nut shell liquid (CNSL). CNSL is a unique source of naturally occurring long-chain hydrocarbon phenols and constitutes about 25% of the cashew weight [1], and 30%–35% of the nut shell weight [1]. A number of methods for the extraction of CNSL from the nut shells are known, but the most common are hot-oil and roasting, in which the CNSL oozes out from the shell [2,3]. Other reported methods include organic solvent extraction [4] and extraction by using supercritical carbon dioxide [5]. The composition of CNSL varies depending on the mode of extraction; natural CNSL (*i.e.*, cold, solvent extracted), for instance, contains approximately 60%–65% anacardic acid, 15%–20% cardol, 10% cardanol, and a few percentage of other phenols and less polar substances [6]. On the other hand, in the technical CNSL extraction method (*i.e.*, heat extracted), the heating process leads to decarboxylation of the anacardic acid to form cardanol, and, typically, the composition of technical CNSL is approximately 60%–65% cardanol, 15%–20% cardol, 10% polymeric material, with the remainder being made up of other substances [7]. The common feature of all components of CNSL is the presence of hydrophobic side chains, differing in the degree of unsaturation, approximately 5%–8% saturated (**a**), 48%–49% monoene (**b**), l6%–17% diene (**c**), and 29%–30% triene (**d**) [8] (Figure 1).

Anacardic acid, which is the major component of natural CNSL, has attracted great research interest due to its biological activities such as antitumor, antioxidant, gastro-protective, and antibiotic. In addition, it has been used as a synthon for the production of a variety of biologically active compounds with increased efficiency, and some of them outperform their corresponding standard material [9]. Besides the biological activities, anacardic acid has recently been found to be a potential candidate as a capping agent for the development of nanomaterials [10,11].

While cardanol and cardol can be separated by distillation [12], the separation of anacardic acid from the mixture is challenging when considering the toxicity properties of cardanol (0.42 mg/L, lethal concentration 50 after 48 h exposures) [13] and the thermolability of anacardic acid combined with its biological activity. The once-thought optimum temperature method of separation by low-pressure distillation also leads to the acid thermal decomposition to cardanol, whereas chromatographic methods are not cost effective [13,14,15]. Even though it is not green, the most common anacardic acid isolation method used for practical applications is the one reported by Paramashivappa *et al.* [16]. In this method, anacardic acid is precipitated from CNSL as calcium anacardate, which, upon treatment with hydrochloric acid, is converted back to anacardic acid. Anacardic acid can also be isolated from the mixture by using supercritical carbon dioxide [17]. Crude anacardic acid is a liquid mixture of four compounds (**1a**–**d**) (Figure 1), differing from each other by the degree of unsaturation of their hydrophobic side chains. The side chain of unsaturated acid can be hydrogenated, resulting in saturated anacardic acid, which is a white crystalline solid. Upon re-crystallization, the white crystalline solid form transparent rod-like crystals, whose single crystal structure was recently reported for the first time [18]. The crystal structure shows that anacardic acid crystallizes into a monoclinic system with strong intramolecular hydrogen bonding interactions. Herein, we review the emerging roles of anacardic acids from CNSL and their derivatives in biological applications. The discussion places strong emphasis on the biological applications of natural anacardic acids and activity-oriented derivatizations into semi-synthetic anacardic acids for the production of improved biologically active compounds.

## 2. Biological Activity of Anacardic Acids

### 2.1. Antibacterial Activity of Anacardic Acids

Natural anacardic acids (**1a**–**d**) have been found to be potent antibacterial relatives to salicyclic acid, although their activity is limited mainly to gram-positive bacteria [19]. For instance, the activity of **1d** possessing an alkyl triene side chain against *S. mutans* (ATCC 25175) and *S. aureus* (ATCC 12598) were 2048 and 64 times more effective than salicylic acid [19], respectively, while anacardic acid **1a** with a saturated alkyl side chain exhibited no activity against *S. mutans* (ATCC 25175) up to a Minimum Inhibition Concentration (MIC) of 800 µg/mL [20]. For comparison purposes, the MIC of natural anacardic acids (**1a**–**d**) against bacteria is presented, along with the MIC numbers of common antibacterial drugs (Table 1). It should be noted that, although anacardic acid **1a** did not show any activity against *S. mutans* (ATCC 25175) up to 800 µg/mL MIC [20], it exhibited potent antibacterial activity against *P. acnes* (ATCC 11827) at MIC of 780 μg/mL [21].

**Table 1:** The minimum inhibition concentrations (MIC) of natural anacardic acids (**1a**–**d**) compared with some common standard antibacterials.

Bacterial	Antibacterial	MIC (µg/mL)	Reference
*S. mutans* (ATCC 25175)	1a	˃800	[20]
	1b	6.25	[20]
	1c	3.13	[20]
	1d	1.56	[20]
	Vancomycin	1	[22]
	Ampicillin	0.15	[22]
*S. aureus* (ATCC 12598)	1a	˃800	[20]
	1b	100	[20]
	1c	25	[20]
	1d	6.25	[20]
	Methicillin	1.56	[23]
	Penicillin G	0.049	[23]
*P. acnes* (ATCC 11827)	1a	0.78	[20]
	1b	0.78	[20]
	1c	0.78	[20]
	1d	0.78	[20]
	Amoxicillin	0.117 (MIC_90_)	[24]
	Penicillin G	0.125 (MIC_90_)	[24]

**Figure 2:**
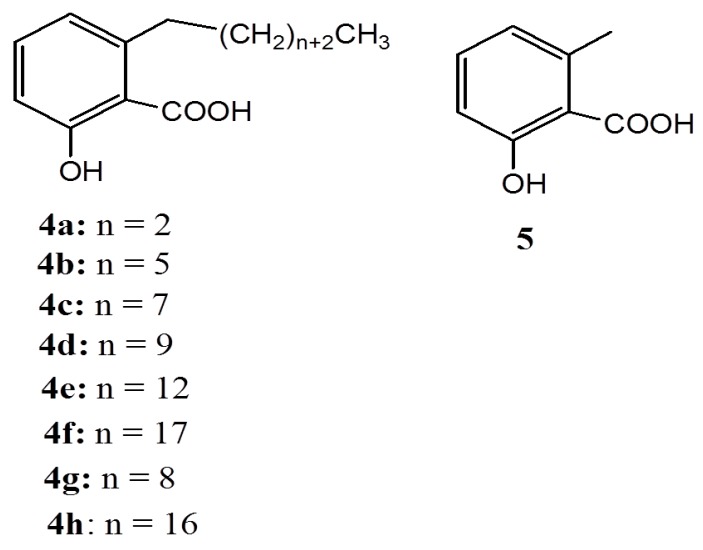
Synthetic anacardic acids, **4** and **5**.

**Figure 3:**
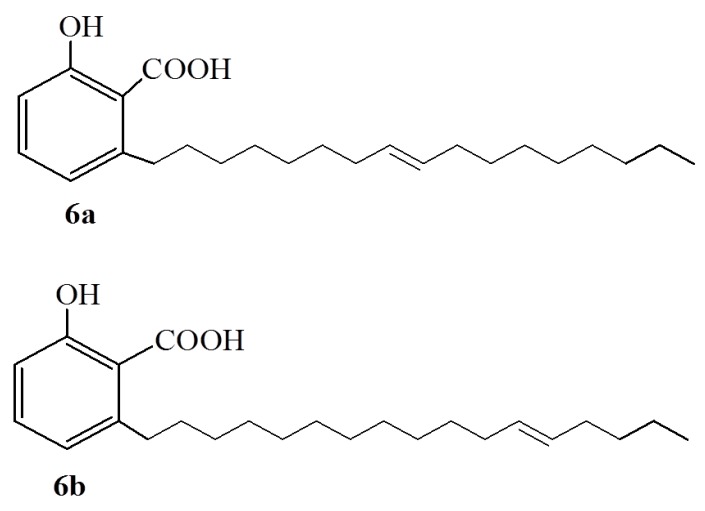
Natural anacardic acid **6a** isolated from ginkobiloba and **6b** isolated from geraniums.

These results show that the linear alkyl side chain and the degree of its unsaturation are both required for enhancing the antibacterial activity against *S. mutans*, whereas the activity against *P. acnes* can be significantly enhanced by a saturated linear alkyl side chain [19,20]. These findings prompted a number of studies [25,26] aimed at gaining more insight into the structure-antibacterial activity relationship (SAR) of anacardic acids. Through these studies, it was later accepted that the optimization of the activity of anacardic acids as antibacterial is possible through semi-synthetic modifications, since the most effective natural anacardic acid (**1d**) is not stable enough for practical application due to the unsaturation of its alkyl side chain [20]. With respect to this, a series of anacardic acids (**4** and **5**) (Figure 2) of **1a** bearing a saturated linear alkyl side chain with different sizes were synthesized, and their activity against various gram-positive bacteria were determined relative to that of **1a**. Anacardic acid **1a** was selected as the standard because of its stability and the easy with which it can be prepared. The results revealed that the length of the alkyl side chain plays an important role in influencing the antibacterial activity but the presence of a double bound is not directly involved. Thus anacardic acid **5** behaves almost similar to salicyclic acid, in that, it exhibits weak and broad antimicrobial activity while **4d** behaves almost like **1d** [20]. Upon a further increase in the length of the alkyl side chain, a decrease in antibacterial activity as anacardic acids **4f** (Figure 3) and **6a** (C24:1 ω^9^) (Figure 3) is observed. Both synthetic **4f** and natural **6a** anacardic acids did not show any antimicrobial activity up to 800 µg/mL MIC [20]. It is worth noting that the natural anacardic acid **6a** was not isolated from cashew nut shells but rather was isolated from ginkobiloba plants [20].

**Figure 4:**
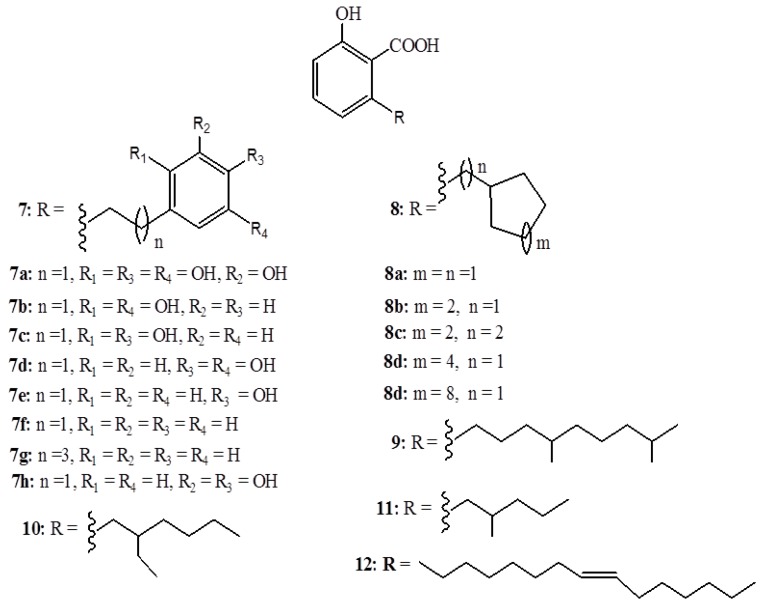
The synthetic anacardic acids (**7**–**12**).

Anacardic acids (**7**–**11**) bearing structurally different side chains (Figure 4) have also been synthesized elsewhere [25] and assayed for activity against *S. mutans* (ATCC 25175) relative to **1d**. Interestingly, anacardic acid **10** (Figure 4), was capable of inhibiting the growth of the *S. mutans* with a minimum inhibition concentration of 780 μg/mL. It is worth noting that this activity is twice that of the most potent natural anacardic acid, **1d**, stressing the importance of a branched side-chain.

A detailed investigation of bactericidal activity against *S. mutans* (ATCC 25175) of various anacardic acids was performed and confirmed by the time-kill curve method to determine their ideal role as anticary agents [26,27]. Among the tested natural anacardic acids **1b**–**d** (Figure 1) and synthetic anacardic acids, **4d** (Figure 2) showed bactericidal activity against this cariogenic bacterium. Excitingly, anacardic acid **1c** was the most effective with a minimum bactericidal concentration (MBC) of 3.31 µg/mL while the MBC for **1b**,**d** and **4d** was 6.25 µg/mL. In addition, anacardic acids **1d** and **4d** exhibited bactericidal activity against methicillin resistant (ATCC 33591 and ATCC 33592) and methicillin susceptible (ATCC 12598 and ATCC 25923) *S. aureus* strains.

A combination study [23], in which anacardic acids were combined with standard antibacterials, revealed a synergistic effect. In this case, synergism was defined as “at least 100-fold (2 log_10_) increase in killing at 48 h by the combination as compared to the most active single compound”.

Apart from synergism, the results obtained were also consistent with the previous reports [21] in showing that the presence of a double bond is not essential in an antibacterial activity increase but is associated with synergism and the nature of the alkyl chain. For instance, a combination of methicillin with anacardic acid **4d**, bearing branched side chain revealed equal potency in synergic activity against methicillin resistant *S. aureus* strains (ATCC 33591) [23] as the combination of methicillin with natural anacardic acid **1d**. Additionally, a combination of **1d** with anethole or linalool [26] was observed to act synergistically against *S. mutans* (ATCC 25175).

Generally, anacardic acids and their derivatives have shown to have potential antibacterial activity and when natural anacardic acids are derivatized, their activity is no longer limited to gram-positive bacteria only. The synergic activity against a variety of resistant strains is also observed upon combining anacardic acids with standard drugs.

### 2.2. Anacardic Acids as Antitumor Agents

Histone acetyl transferases (HATs) are a group of enzymes that catalyze acetylation of histones and, thereby, regulate the gene expression. Histone acetyl transferases (HATs) are divided into GNAT (GCN5-related *N*-acetyl transferase), MYST (Moz, Ybf2/Sas3, and Tip60), and p300/CBP (CREB-binding protein) families. The CBP (CREB binding protein)/p300, is among the most studied transcriptional co-activators, as it plays a significant role in a variety of cellular processes and, therefore, their dysfunction is associated with several serious human diseases such as cancer, diabetes, viral infection and asthma [28].

**Figure 5:**
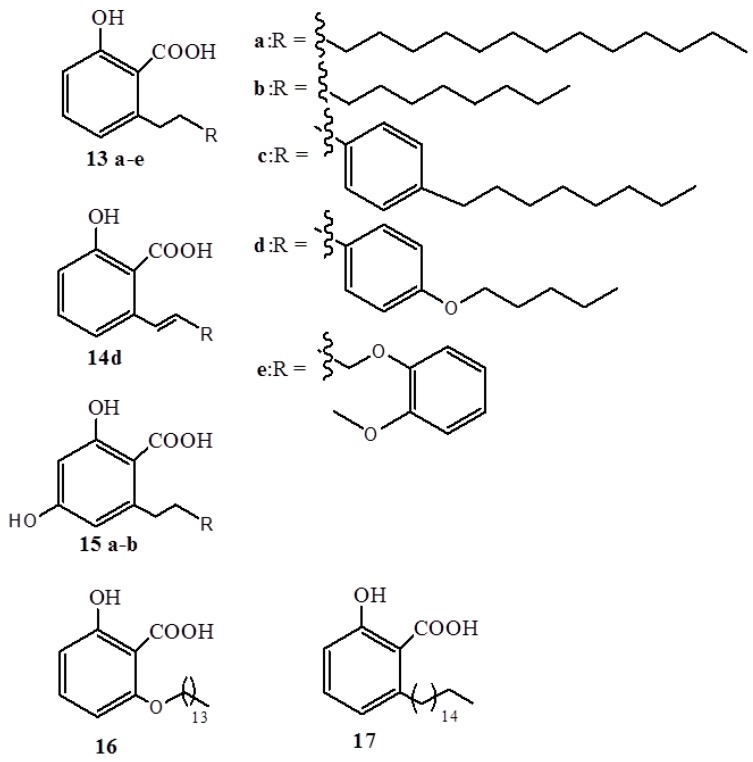
Synthetic anacardic acids **13**–**17**.

A mixture of anacardic acids (**1a**–**d**) inhibits HAT activity of p300 and P300/CBP-associated factor (PCAF) *in vitro* [29]. Remarkably, the hydrogenation of the mixture to saturated acid **1a** did not alter the inhibitory activity, hence eliminating the importance of unsaturation in the inhibition mechanism. In an effort to optimize HAT inhibitory potency using molecular modeling techniques, a binding model of anacardic acids in the PCAF active site was proposed [29]. It was found that in the course of inhibition, the salicylate moiety, which mimics the pyrophosphate group of CoA, forms hydrogen bond networks with various groups like the amide nitrogens of V582, G584, G586, and T587, the hydroxyl of T587, and the water molecule w6. On the other hand, the alkyl chain is observed to stay in the hydrophobic pantothenic acid binding pocket. Based on this model, a series of derivatives were synthesized (**13**–**17**) (Figure 5) and tested, whereby, **14d** was found to be twofold superior to **1a** in the inhibition of HAT, PCAF, and histone H4 acetylation of human liver carcinoma cell line (HepG2) cells [30]. It has been found that inhibition of PCAF by **14d** leads to a decreased proliferation, induced apoptosis, and broken resistance to DNA damage-induced cell death in BCR-ABL-Expressing Cells [31]. Anacardic acids also efficiently inhibit Tip60, the MYST family of HATs *in vivo* with 50% inhibitory concentration (IC_50_) value of 9 µM, that is, they sensitize cells to the cytotoxic effects of ionizing radiation and hinder acetylation and activation of the ATM protein kinase by DNA damage in HeLa cells [32]. In addition, anacardic acids reduce the autophosphorylation of serine 2056, thereby hindering the activation of DNA–PKcs [32]. The radio-sensitizing property of anacardic acids was also studied in detail in V79, SW1573, and U2OS cells; however, a significant effect at a low-toxic concentration of 100 µM was only observed in U2OS cells [33].

In addition, anacardic acids were found to cause the hypoacetylation of histone H3 at K9 as well as the down regulation of active genes [34]. Although this happens only with a minimal net reduction of HAT activity, it is linked to the reversibility of the process. Other researchers have also explained the mechanisms by which anacardic acids inhibit HAT activities and thus control many kinds of cancer. For instance, Sung *et al*. [35] revealed that anacardic acids mediate antitumor activity through inhibition of nuclear factor NF-κB activation by various stimuli such as carcinogens, growth factors, tumor promoters, and ionizing radiation. It has been reported that activated NF-κB in the nucleus binds to κB recognition sites and to HAT families such as p300/CBP and PCAF [36] and, in doing so, facilitates acetylation of lysine residue in histone H4 and the opening of the chromatin structure [37].

Furthermore, for activity against prostate cancer, anacardic acids inhibit the proliferation and apoptosis of prostate cancer cells and hence induces cell cycle arrest such that it activates P53 signaling, inhibits NFκB, IkBa kinase and androgen receptors (AR), thereby suppressing p300 [38]. Anacardic acid (**1a**) is found to inhibit angiogenesis by targeting steroid receptor coactivator (Src), focal adhesion kinase (FAK), and the Rho family of GTPase signaling pathways [39].

It has also been found that anacardic acids selectively promote the activity of Aurora kinase A *in silico* in the presence of both AURA and AURB and, thus, mediates phosphorylation of histone H3 [40]. Aurora kinase (A, B & C) is the family of enzymes which play a crucial role in cell division, and, therefore, their dysfunction can lead to an uncontrolled cell proliferation and is associated with many kinds of cancer. In the case of breast cancer, while anacardic acids (**1a**–**d**) are active cytotoxic agents against BT-20 breast carcinoma cells [41], anacardic acid **6b** (C24:1 ω^5^) from geraniums (pelargonium × hortorum) inhibits the proliferation of estrogen receptor α-expressing breast cancer cell lines, estrogen response element interaction, and the transcription of estrogen response target genes [42]. In addition, anacardic acids (**1a**) are found to intrinsically induce apoptosis in the A549 human lung adenocarcinoma cells [43], and, in combination with vitamin C and an anticancer agent, mitoxantrone, they synergically enhance the cytotoxicity of melanoma cell lines with efficient protection of normal cells against mitoxantrone [44].

Based on the findings from several reported studies, anacardic acids have recently been considered among the potential antitumor chemicals which require more research to further derivatize them in order to unlock more of their potentials against cancer as well as to contribute towards establishing the mechanism of their anticancer activity by a structure activity relationship (SAR).

### 2.3. Anacardic Acidsas Antioxidants

Antioxidants (“free radical scavengers”) are chemicals that interact with and stabilize free radicals in chain reaction mechanisms, making them non-reactive under normal conditions, thereby inhibiting the propagation of chain reactions. Antioxidants, therefore, inhibit the propagation of chain reactions by stabilizing the radical species, thus preventing them from causing damage to the body cells.

As antioxidants, anacardic acids **1a** and **6b** have demonstrated inhibitory potentials against both potato lipoxygenase and ovine prostaglandin endoperoxide synthase with almost the same potency of approximate IC_50_ of 6 and 27 µM, respectively [45]. Moreover, monoene anacardic acid **1b** has been found to competitively inhibit the soybean lipoxygenase-1 (EC 1.13.11.12, type 1), which catalyzes the linoleic acid peroxidation with IC_50_ of 6.8 μΜ [46,47,48,49,50,51]. On the other hand, anacardic acids **1a**, **1c**, **1d**, cardanol **2b**, and salicyclic acid were tested for activity against this enzyme under the same conditions. The results revealed that anacardic acids **1b** and **1d** bearing 1(Z), 4(Z) pentadiene systems were oxidized as substrates at low concentrations [47,48], whereas at higher concentrations (>40 μΜ), they both inhibited the peroxidation [47]. The fact that salicylic acid did not show any inhibitory activity gives an indirect proof that the bisallylic methylene groups of the alkyl side chain is essential and, thus, the activity increases with alkyl chain length. Conversely, compound **4g** (Figure 2), bearing an alkyl side chain with 12 carbons is slightly less potent relative to **5**, which possesses a methyl side chain [51].

It has also been noted that the hydrophobic side chain alone is not sufficient in inhibiting the peroxidation of linoleic acid since cardanol, which possess the same alkyl side chain as anacardic acid, was completely inactive. This observation implies that the salicylic acid moiety in the anacardic acid is vital for its activity [46]. Although the double bond in the side chain of anacardic acids participate in increasing their inhibition potency, the stereochemistry of the side chain has a role in the anti-peroxidation potency. This is confirmed from the observation that saturated anacardic acid **1a** shows anti-peroxidation activity (IC_50_ value 14.4 μΜ), which is twofold inferior to that of **1b** (6.8 μΜ) [46,47]. In addition, *E*-isomer **12** (Figure 4) behaved similarly to its Z-isomer **1b** [51].

The ability of anacardic acids to form metal chelation [3] is of importance in assessing the antioxidant behavior of anacardic acids. Metal chelation reduces the concentrations of the transition metal content of the enzyme-catalyzing lipid peroxidation and, hence, affects the peroxidation. On this front, workers [50] have suggested that lipoxygenase inhibition by anacardic acids starts with ion chelation in the active site of the enzyme by the hydrophilic portion of anacardic acids, followed by a slow interaction of the hydrophobic tail with the *C*-terminal domain containing the iron [50]. This suggests that the head-tail structural optimization by synthetic methods is worthwhile and, in response, compounds **4g**–**h** (Figure 2), **7a**–**c**, **7h**, as well as **9**–**10** (Figure 4), have been synthesized and tested for their activity against lipoxygenase. Compounds **9** and **10** were found to be potent lipoxygenase inhibitors implying that the shape and size of the side chain are important parameters. This observation agrees well with the trends observed in antibacterial applications of anacardic acids [25,26,27].

Anacardic acid **1d** also inhibited the generation of superoxide and uric acid by xanthine oxidase in a noncompetitive fashion without notable radical-scavenging activity [9,52,53]. It has been suggested that a higher affinity shown by anacardic acids relative to that of salicylic acid is due to the presence of an alkenyl side chain which interacts with the hydrophobic site of xanthine oxidase and thus enhances the inhibition initiated by salicylic acid moiety of anacardic acids through binding to the Mo-pterin domain of the enzyme [53]. The metal chelation phenomenon explains why cardanol exhibited no anti-oxidant activity against the xanthine oxidase [52].

In another study [25], anacardic acid **1d** inhibited oxygen consumption of *M. luteus* ATCC 4698 and *P. aeruginosa* IFO 3080 when the suspensions prepared from the cells of these bacteria were incubated with the anacardic acid. Moreover, anacardic acids **1a**–**d** inhibited *M. luteus* and *P. aeruginosa* NADH oxidation by a membrane fraction prepared from the same bacterial cells, while salicylic acid, cardanol **2d**, did not exhibit respiratory inhibition [25]. Likewise, these results indicate that respiratory inhibition activity requires the presence of both salicylic acid moiety and the alkyl side chain, and the degree of unsaturation is not directly linked to the activity. It should be noted that anacardic acids (**1a**–**d**) have already shown a dose-dependent inhibitory activity of oxidation of l-DOPA by the mushroom tyrosinase [25]. Analysis made by Kubo *et al.* [21,25] indicated that the antibacterial activity of anacardic acid is due to mainly physical disruption of the bacterial cell membrane in which anacardic acids act primarily as surfactants and, for activity to be attained, a proper balance between hydrophilic and hydrophobic parts of the anacardic acid is essential [21].

Anacardic acid **1a** also outperformed the standard uncoupler, 2,4-dinitrophenol (DNP) in its efficiency on the uncoupling action of oxidative phosphorylation of isolated rate mitochondria using succinate as a substrate. Unlike DNP in parallel with the uncoupling effect, **1a** also showed a small inhibition of the respiration rate. The inhibition of the respiration rate shown by **1a** is smaller relative to that reported onarachidonic acid [54], implying that the alkyl side chain has an influence on the uncoupling effect [55]. It has also been found that oral treatment with anacardic acids improve antioxidant activity of glutathione peroxidase (GPx), glutathione reductase (GR), glutathione *S*-transferase (GST), and catalase (CAT), enzymes of BALB/c mice lung damaged following exposure of animals to diesel exhaust particulate [56]. The most effective dose of anacardic acids, 50 mg/kg, decreased levels of neutrophils and tumor necrosis factor (TNF-α) in the lung parenchyma and in the broncho alveolar lavage fluid (BALF) supernatant, respectively [56].

Furthermore, anacardic acids have showed gastro-protective effects against gastric mucosal damage induced by ethanol through oxidative mechanisms in which anacardic acids restore oxidative activity of superoxide dismutase and catalyze enzymes and nitric oxide levels, thereby reducing undesirable, highly-reactive free superoxide radicals in the cell membrane [57]. Anacardic acid **1a** is observed to strongly inhibit α-glucosidase, while anacardic acid **1d** is a potent inhibitor of aldose reductase and invertase, thereby vital for control of obesity and diabetes [58]. Anacardic acids also inhibit a number of other enzymes and there is great potential to unravel its full potential as an antioxidant.

## 3. Anacardic Acids as Synthons for Synthesis of Biologically Active Products

Although anacardic acids have been reported as bioactive molecules, their activities are not potent enough as drug candidates and are mainly limited to gram-positive bacteria. The instability of the most active anacardic acid, **1d**, is due to the presence of non-isoprenoid long alkenyl side chain moiety that renders this molecule less useful for therapeutic purposes. Todate, a number of strategies have been reported, aiming at optimizing the bioactivity of anacardic acids including the combining of anacardic acids with standard antibacterial compounds along with synthesized anacardic acid derivatives. The proved utility of the easily accessible, environmentally benign, naturally occurring anacardic acids as potential starting material for the development of potent bioactive compounds has attracted a number of researchers’ interest.

**Figure 6:**
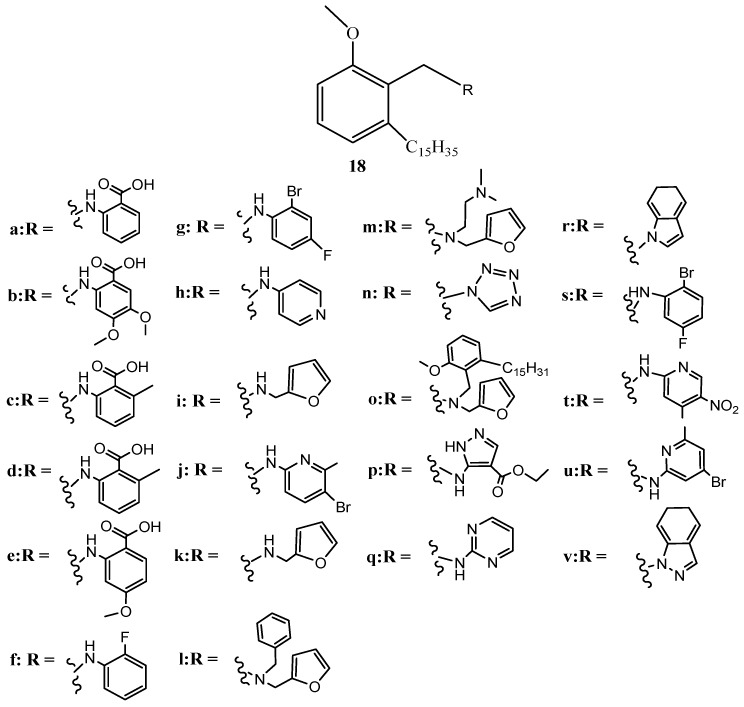
The benzyl amine derivatives of anacardic acid (**18a**–**v**).

**Figure 7:**
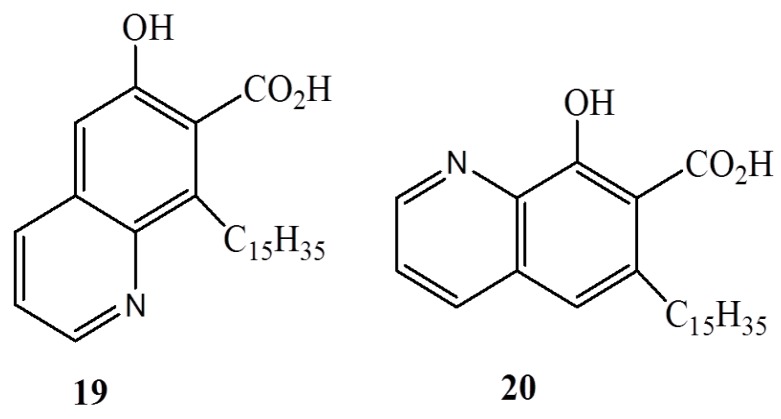
The quinolines **19** and **20**.

A series of benzyl amine derivatives of anacardic acid (**18a**–**v**) [59] have been developed (Figure 6) from anacardic acids and assayed for activity against both gram-negative (*E. coli* and *P. aeruginosa*) and gram-positive bacteria (*S. aureus* and *S. pyogens*) and compared with standard ampicillin, chloramphenicol, ciprofloxacin, and norfloxacin [59]. The results revealed that none of the synthesized anacardic acids was superior to standard antibiotics against all tested bacteria. The most potent anacardic acid, **18q**, showed activity comparable with standard ciprofloxacin on *S. aureus*, but was inferior against *P. aeruginosa*. Among the effective compounds were anacardic acids **18n** and **18k**, and their bioactivity against *P. aeruginosa* and *S. pyogens*, respectively, were comparable to ampicillin, whereas testing of activities against *E. coli* demonstrated that substituted anthranilic acids and pyridines outperformed other benzyl amines. Quinolines **19** and **20**, which are well known anti-bacterial agents, have also been prepared using anacardic acids as synthons (Figure 7) [60].

**Figure 8:**
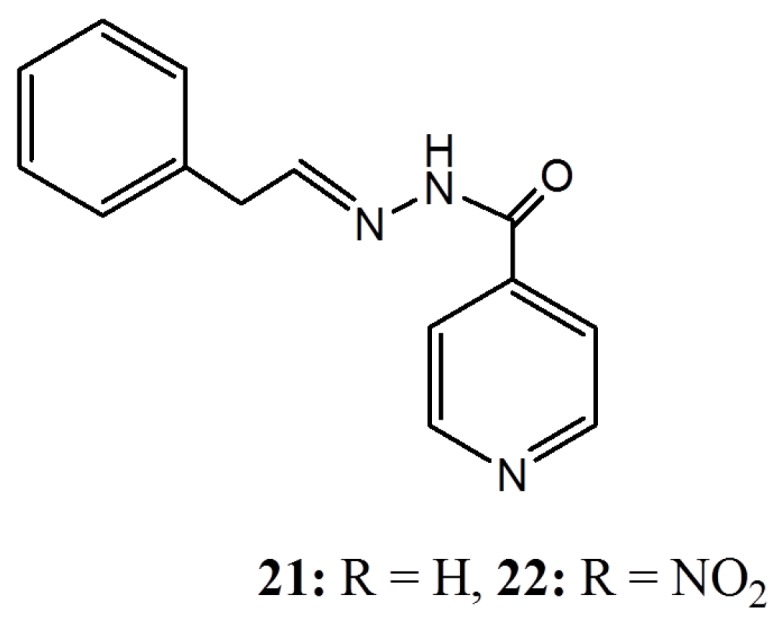
The isonicotinoylhydrazones **21** and **22**.

Isonicotinoylhydrazones **21** and **22** (Figure 8) have similarly been synthesized from anacardic acid and found to be active against *Mycobacterium smegmatis* mc^2^155 while natural anacardic acid is inactive [61]. Although compounds **21** and **22** showed inferior activity relative to the isoniazid (INH), their potency increased considerably when used in combination with INH. Compounds **21** and **22** also showed some inhibitory activity against pathogenic strain *M. tuberculosis* H37Rv. The activities of **21** and **22** against *Mycobacteria* suggest the involvement of possibly a complete different biological target compared to earlier described antibacterial activities, as compounds **21** and **22** do not require the hydroxyl and acid groups for their activities.

**Figure 9:**
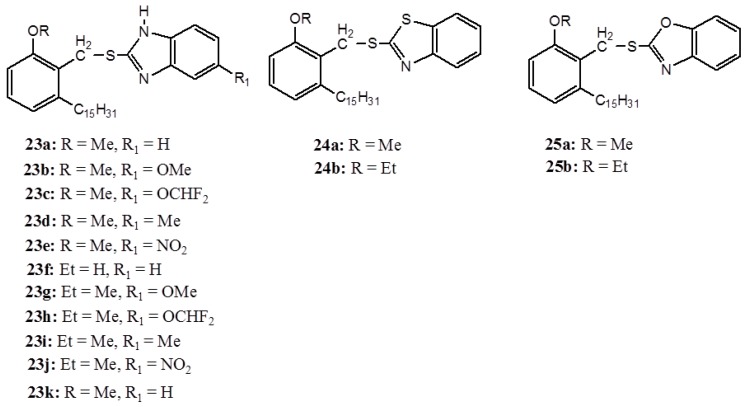
The benzimidazole (**23a**–**k**), benzothiazole (**24a**–**b**) and benzoxazole (**25a**–**b**) derivatives.

In another development, anacardic acids have been used as synthons for synthesis of a series of cyclooxygenase-2 (COX-2) inhibitors (**23**–**25**) (Figure 9) [62]. Although the overall inhibition activity of these complexes are weaker than the standard rofecoxib, strikingly, compounds **23f** and **24a** showed high selectivity and are efficient enough to be applied against cyclooxygenase-1 (COX-1).

**Figure 10:**
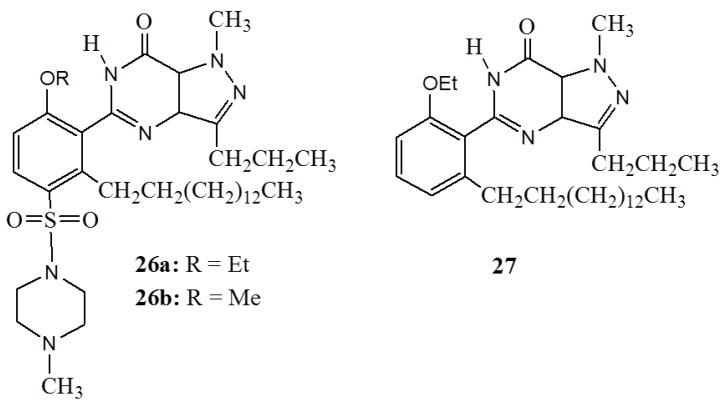
The sildenafil analogues **26** and **27**.

Sildenafil is among the potent inhibitors of phosphodiesterase enzyme type 5 (PDE5) that prevents the inactivation of the intracellular second messengers cyclic adenosine monophosphate (cAMP) [63]. For that, it is an oral active drug in the treatment of male erectile dysfunction, although it is associated with several side effects. Sildenafil analogues, which are derivatives of anacardic acids (**26**,**27**) (Figure 10) have been developed from anacardic acid and assayed on partially purified PDE_5_ enzyme [64]. Even though the biological activity of anacardic acids and their derivatives have been associated with the presence of hydrophobic side chains which are not found in the salicylic acid analogs; in this particular case, an alkyl side chain showed a negative effect as all new complexes (**26**,**27**) had inferior activity compared to the standard sildenafil compounds. In comparison, the less potent activity by complex **27** relative to the complexes **26a**–**b** signifies the importance of the *N*-methylpiperazinesulfonamide group in their biological activity.

**Figure 11:**
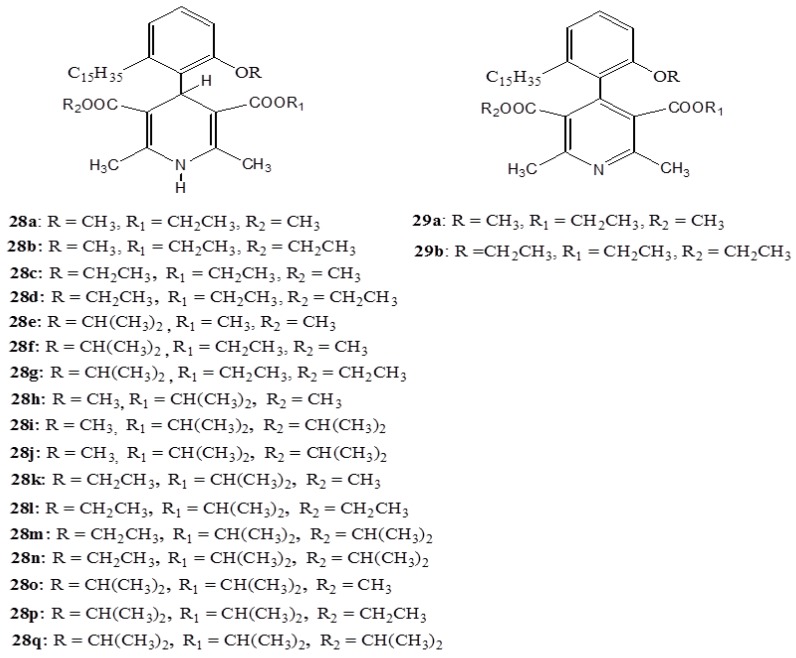
The dihydropyridine derivatives **28** and **29**.

The negative effect of the hydrophobic pentadecyl side chain has been experienced in the blocking affinity of a series of novel dihydropyridine (DHP) derivatives [dialkyl 1,4-dihydro-4-(2'-alkoxy-6'-pentadecylphenyl)-2,6-dimethyl-3,5 pyridine dicarboxylates] (**28**,**29**) (Figure 11) developed from anacardic acid and tested [65]. Although to a lesser extent relative to the classical nifedipine complex, the developed DHP-derivatives showed the tendency to block L-type calcium channels transiently expressed in *t*SA-201 cells and where, in some cases, the T-type cells were also blocked. The higher efficacy of these compounds found to be favored by the increasing the size of the alkoxy group on 4-phenyl ring and ester substituent in the 3,5 positions. Even if the more potent (**28k**) exhibited irreversible action different from the standard nifedipinein, the complex (**28k**) resembles nifedipinein in many aspects. For instance, similar to the nifedipine, this compound accelerated the time course of current decay and mediated a robust shift in the midpoint of the steady-state inactivation curve toward more hyperpolarized potentials [65].

**Figure 12:**
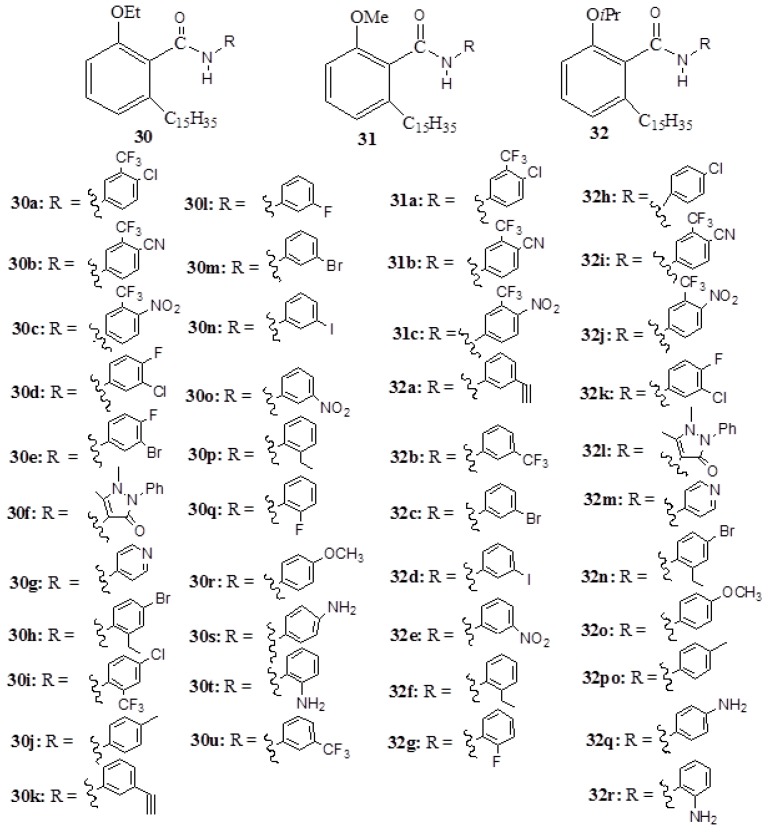
The amide derivatives (**30**–**32**).

Although anacardic acids were reported to inhibit HATs [28,29,30,31,32], they are not specific to a particular group. Interestingly, amide derivatives (**30**–**32**) (Figure 12), which have been prepared from anacardic acid synthons, are selective activators of p300 HAT activity, albeit with varying degrees of activation with some of them being cell permeable [31,66,67,68]. The salicylic acid analogy of amide complexes have also been prepared to elucidate the structural basis and the mechanism in which amide complexes mediate the activation of p300 HAT activity. The fact that only salicylic acid derivatives with isopropyl functionality could stimulate the p300 HAT activity suggests that the presence of a pentadecyl side chain is not necessary. A detailed analysis by Surface-enhanced Raman spectroscopy (SERS) revealed that, in the course of activation, the activator complexes bind to the p300 and cause changes in the basic structure of the enzyme [67].

**Figure 13:**
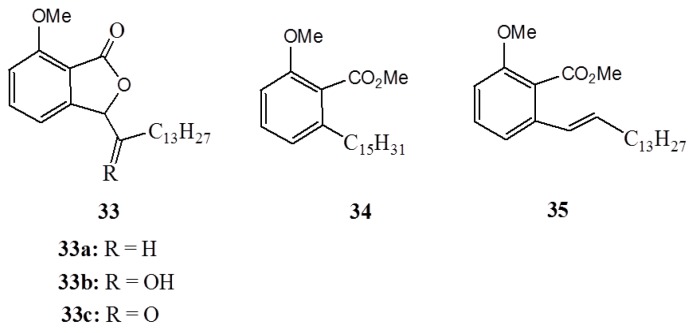
The cytotoxic compounds **33**–**35**.

Isobenzofuranones (**33a**–**c**) and their acyclic precursors (**34**,**35**) (Figure 13) have also been developed starting from anacardic acid and evaluated for their antiproliferative activity against HL-60 leukemia, SF295 glioblastoma and MDA-MB435 melanoma human cancer cell lines, and peripheral blood mononuclear cells (PBMC) relative to doxorubicin [29]. The results showed that complex **35** and isobenfuranones (**33a**–**c**) are active cytotoxic compounds, however, to a lesser extent than doxorubicin. The detailed analysis of the mechanism by which the cytotoxic compounds work revealed that the activity is the result of the apoptosis or necrosis induction which leads to the DNA degradation [29].

**Figure 14:**
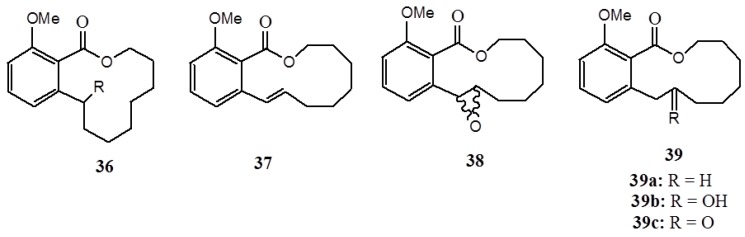
The macrolides (**36**–**39**).

Antiproliferative activity against miclymphoblastic leukemia (CEM), breast cell line (MCF-7), human colon (HCT-8), and murine skin (B16) of macrolides (**36**–**39**) (Figure 14) synthesized from anacardic acid was also accomplished relative to the doxorubicin [69]. Likewise, the standard doxorubicin outperformed all synthetic compounds. While complex **36b** and **37** showed cytotoxicity activity against all tested cell lines, compound **36a** was active only against mammalian cells and **38** was active only against HCT8 and MCF-7 cells. These synthesized macrolides were also assayed against artemiasalina in which, remarkably, compound **36a** outperformed all standard active controls (lapachol and potassium dichromate), while compound **39** displayed significant activity [69]. Compounds **36b**, **37**, and **38** were inactive at a concentration of up to 200 ppm.

**Figure 15:**
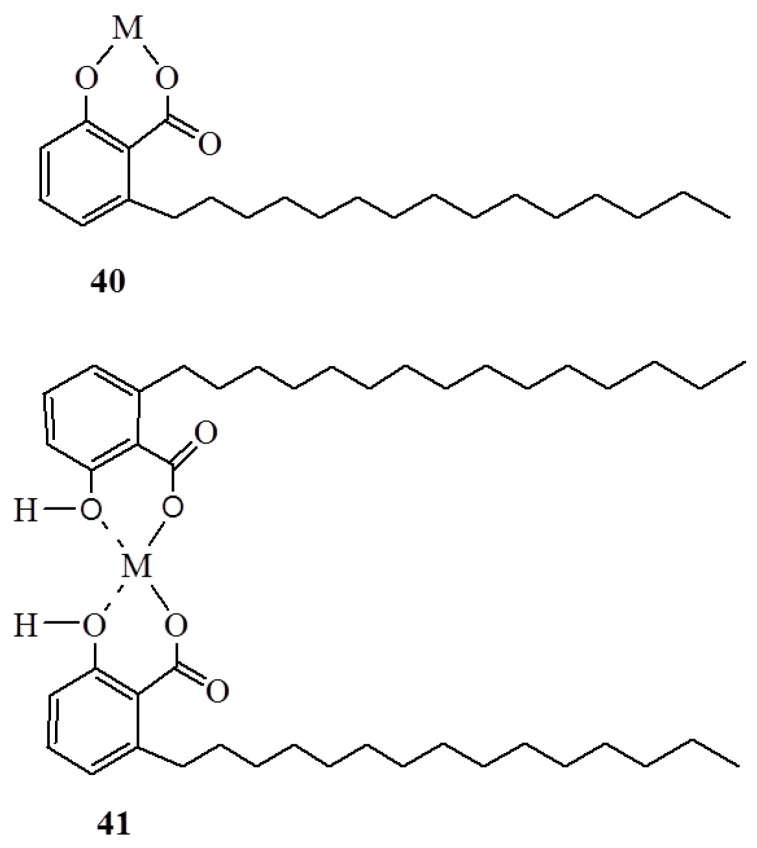
The metal complexes of anacardic acids **40** and **41**.

In general, the structure of anacardic acids produce chemical properties which make them potential precursors for developing a diverse number of valuable products. The combination of phenolic and carboxylic acid groups renders anacardic acid a powerful chelating ability to metal ions and/or metal complexes. For example, the anacardic acid **1a** (Figure 1) has shown a great ability to form lipophilic metal derivatives in ratios of both 2:1 and 1:1 (**40**,**41**) (Figure 15) with a high degree of selectivity among the first row transition metals. The chelating affinity of **1a** followed the trend: Fe^2+^ > Cu^2+^ > Zn^2+^ > Ni^2+^ = Co^2+^ = Mn^2+^ for the **2**:**l** derivatives [70]. The ability of anacardic acid to form metal chelation with important metals like Fe^2+^ and Cu^2+^ can provide a rational explanation of the wide spectrum of biological activity by anacardic acids.

Pyridine adduct of copper (II) anacardate has recently been reported [18] as shiny green, plate-like crystals. The strong antiferromagnetic coupling between metal centers detected by magnetic and EPR studies suggested the dimeric structure of the pyridine adduct. The blue powdery monomeric pyridine adduct of copper (II) anacardate was also isolated as a minor product and detected by magnetic as well as by EPR studies [18]. The structure of the crystals was unambiguously confirmed by X-ray diffraction studies as dimeric complex, [Cu(Anac)_2_(py)]_2_, with a configuration similar to that reported for copper(II) salicylate analogs, in which four molecules of anacardic acid, through their carboxylate bridges, bind two Cu(II) atoms, and phenolic groups are not involved in the coordination [18]. In the semi-octahedral geometry formed by each copper atom, the equatorial and axial positions are occupied by four carboxylate oxygen atoms of two anacardic acids and the nitrogen atom of pyridine, respectively. This mode of coordination is, however, different from the one proposed for metal ions [70] (Figure 15), in which both carboxylate and phenolic bridges participate in the coordination. The former marks the first report for such metal complexes of anacardic acid to be fully characterized, and this should be key towards understanding the biological activity of anacardic acid and its derivatives.

## 4. Conclusions

Cashew nut shells, which are otherwise agrowastes of cashew nut processing factories, upon treatment and refinery, produce cashew nut shell liquid (CNSL),which is a unique source of naturally occurring long-chain hydrocarbon phenols. Impressive research work has been performed towards transforming the agrowastes (cashew nut shells) into valuable biologically active and useful products. Of particular interest in this case is that one of the CNSL components, (*i.e.*, anacardic acids) exhibits multifunctional roles and has been used as a bioactive agent. The extract has also been used as a synthon for the synthesis of various analogues for a variety of possible biological applications. As bioactive phytochemicals, the structure activity relationship has been fully explored and it is found that neither the double bond nor the stereochemistry of the side chain alone is a necessary condition, even if they synergistically participate in influencing the activity. On the contrary, the length of the alkyl side chain and the hydrophilicity of the anacardic acid head group play an important role in its various biological activities. This head-tail structural optimization has consequently led to a number of anacardic acid derivatives being developed. Some of these developed derivatives outperformed the natural anacardic acids and/or standard complexes for a particular application. In the case of enzyme inhibition, a variety of mechanisms have been proposed. For instance, it has been proposed that anacardic acids inhibit HAT through inhibition of nuclear factor NF-κB activation by various stimuli such as carcinogens, growth factors, tumor promoters, and ionizing radiation. The ability of anacardic acids to form metal chelation was also noted in order to reduce the concentration of the transition metal content of the enzyme and hence providing a clue as to why anacardic acids have various biological activities. Anacardic acids have also been utilized for the development of diverse valuable products such as the production of bioactive complexes, nanomaterials, and metal ions and/or metal complexes. The combination of this information demands more work to be done on the potential of anacardic acids in biological applications in order to come up with products and ways in which anacardic acids and their derivatives can not only inhibit various health problems but can also cure and prevent various diseases at different stages. In addition, as oil supplies dwindle and its price rises, anacardic acids from agrowaste can replace petroleum based chemicals and products in our daily use.
